# Improving the Laboratory Diagnosis of M-like Variants Related to Alpha1-Antitrypsin Deficiency

**DOI:** 10.3390/ijms23179859

**Published:** 2022-08-30

**Authors:** Valentina Barzon, Stefania Ottaviani, Alice Maria Balderacchi, Alessandra Corino, Davide Piloni, Giulia Accordino, Manuela Coretti, Francesca Mariani, Angelo Guido Corsico, Ilaria Ferrarotti

**Affiliations:** 1 Centre for Diagnosis of Inherited Alpha-1 Antitrypsin Deficiency, Department of Internal Medicine and Therapeutics, University of Pavia, 27100 Pavia, Italy; 2 Centre for Diagnosis of Inherited Alpha-1 Antitrypsin Deficiency, Laboratory of Biochemistry and Genetics, Pulmonology Unit, Fondazione IRCCS Policlinico San Matteo, 27100 Pavia, Italy

**Keywords:** alpha1-antitrypsin, *SERPINA1* gene, rare pathogenic variants, M_wurzburg_, M_whitstable_, laboratory diagnosis

## Abstract

Alpha1-antitrypsin (AAT) is a serine protease inhibitor that is encoded by the highly polymorphic *SERPINA1* gene. Mutations in this gene can lead to AAT deficiency (AATD), which is associated with an increased risk of lung and/or liver disease. On the basis of electrophoretic migration, AAT variants are named with capital letters; M (medium) signifies the normal protein. Among pathological variants, the M-like ones represent a heterogeneous group of rare allelic variants that exhibit the same electrophoretic pattern as the M wild-type protein, which makes them difficult to detect with routine methods. In order to avoid their misdiagnosis, the present study defines and validates effective methods for the detection of two pathogenic M-like variants, M_wurzburg_ and M_whitstable_. Comparison of protein phenotypes using isoelectric focusing of samples that presented the M_wurzburg_ variant, as revealed by exons 5 sequencing, identified a particular electrophoretic pattern amenable to the M_wurzburg_ protein. The specific phenotyping pattern was retrospectively validated, thus enabling the detection of 16 patients with M_wurzburg_ variant among the subjects already tested but not sequenced according to our diagnostic algorithm. The M_whitstable_ allele was detected by intron 4 sequencing of SERPINA1 gene. M_wurzburg_ and M_whitstable_ are often misdiagnosed and the introduction of diagnostic improvements can help the clinical management, especially in patients with established lung disease without any other reported risk factors.

## 1. Introduction

Alpha1-antitrypsin (AAT) is a serine protease inhibitor mainly produced in the liver and, to a minor extent, by neutrophils, monocytes, and epithelial cells in the lung and gut. The key function of AAT is the regulation of the proteolytic effects of neutrophil elastase (NE) in the lungs [1]. AAT is encoded by the highly polymorphic *SERPINA1* gene (or Protease Inhibitor (PI) system) located on the long arm of chromosome 14 (14q32.13). The *SERPINA1* gene is organized into four coding and three non-coding exons and spans over 12.2 kb. The encoded protein includes 394 amino acids, with the active site of the enzyme inhibitor at methionine 358. Low AAT serum levels can lead to Alpha1-antitrypsin deficiency (AATD) (OMIM #613490; https://www.ncbi.nlm.nih.gov/omim/; accessed on 3 August 2022), a disorder caused by variations in the *SERPINA1* gene, inherited in an autosomal recessive pattern with co-dominant expression of alleles. AATD is related to an increased risk of developing early-onset emphysema and/or chronic liver disease [2].

Over 120 variants of AAT have been described [3]. The wild-type allele is referred to as the M allele; the Z (p.E366K c.1096G>A rs28929474) and S (p.E288V c.863A>T rs17580) alleles are the most frequent variations that lead to AATD, but several other pathogenic variants, usually referred to as “rare”, have been identified [4]. An updated list of allelic variants was recently published [3]. Due to the different molecular mechanisms linked to each variant, wide variations are seen in the incidence and severity of lung and liver disease.

Rare variants are usually thought to have a low prevalence, but many of them are actually rarely investigated and under-diagnosed [5]. One reason could be the difficulty in identifying them using routine analysis. Many rare variants can only be detected by molecular methods, such as PCR sequencing, that are not available in all diagnostic laboratories. To date, there is no universally-established algorithm used by laboratories for the detection of AATD patients. The algorithms applied in different countries depend on diverse criteria based on the availability of diagnostic facilities and expertise, country-specific indications, and the occurrence of variants [6].

The “classic” nomenclature of AAT variants was established based on electrophoretic migration of AAT serum before the encoding SERPINA1 gene was identified [7]. AAT variants were initially named on the basis of their migration velocity in starch-gel electrophoresis as M (medium), S (slow), F (fast), or Z (very slow). Subsequently, the other alleles were designated with a letter A–L or N–Z depending on their proximal or distal location, respectively, to the M protein band. When a new variant is identified, the birthplace of the index case is added to the letter that corresponds to the IEF migration.

M-like variants (M_malton_, M_procida_, M_heerlen_) are a heterogeneous group of rare AAT mutations that display the same electrophoretic pattern as the M wild-type protein.

This paper focuses on two M-like variants, namely M_wurzburg_ [8] and M_whitstable_ [9], and the diagnostic procedures aimed at their identification. Both alleles are classified as M-like variants because their isoelectric focusing (IEF) pattern is easily confused with the normal M protein. Previously documented M_wurzburg_ electrophoretic patterns [10] are not replicable. The M_wurzburg_ protein (p.P393S c.1177C>T rs61761869; https://www.ncbi.nlm.nih.gov/snp/; accessed on 3 August 2022) is an M-like variant forming ordered polymers that are retained as inclusions within the endoplasmic reticulum (ER) of hepatocytes. The M_wurzburg_ variant was first identified in a heterozygous individual with serum AAT deficiency but no evidence of lung or liver disease [8]. The M_whitstable_ variant consists of a 26-bp deletion with an associated insertion of two deoxyguanosine residues in intron 4. This variant usually arises on the normal M2 allele. The M_whitstable_ mutation was first detected in two related Z heterozygous subjects [9].

## 2. Results

### 2.1. M_wurzburg_

**Figure 1:**
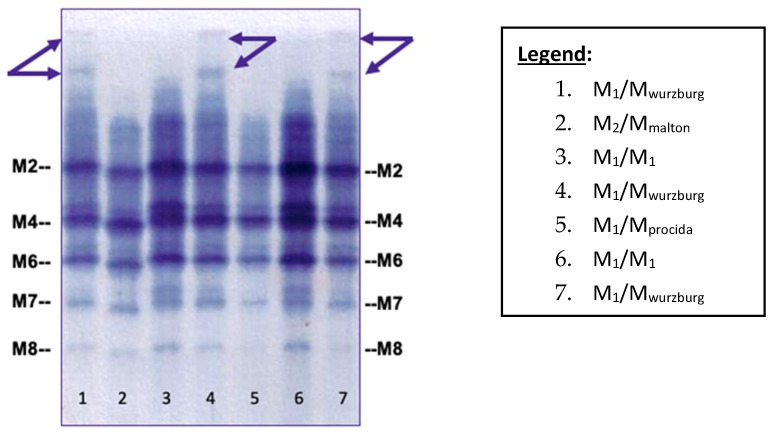
IEF pattern of M-like variants obtained by Sebia Hydrasys^®^ System on DBS samples. M2, M4, M6, M7, and M8 represent the different M isoforms [11,12]. (Arrows show the specific pattern of M_wurzburg_).

We carried out a retrospective review of our diagnostic phenotyping results from March 2014 to January 2019 in all samples carrying the M_wurzburg_ allele (76 out of 5916) as revealed by exon 5 sequencing of the *SERPINA1* gene, which was performed according to the recently described diagnostic algorithm [6]. Direct comparison identified a particular electrophoretic pattern amenable to the M_wurzburg_ protein. Interestingly, the specific IEF pattern was detectable in both serum and dried blood spot (DBS) samples. The specific IEF bands amenable to the M_wurzburg_ protein were clearly visible at the top of the gel, as shown by the arrows in Figure 1.

Conversely, M-like variants such as M_malton_ and M_procida_ are not associated with a particular IEF pattern, but are very similar to the normal M protein pattern (Figure 1, Lanes 2 and 5).

**Figure 2:**
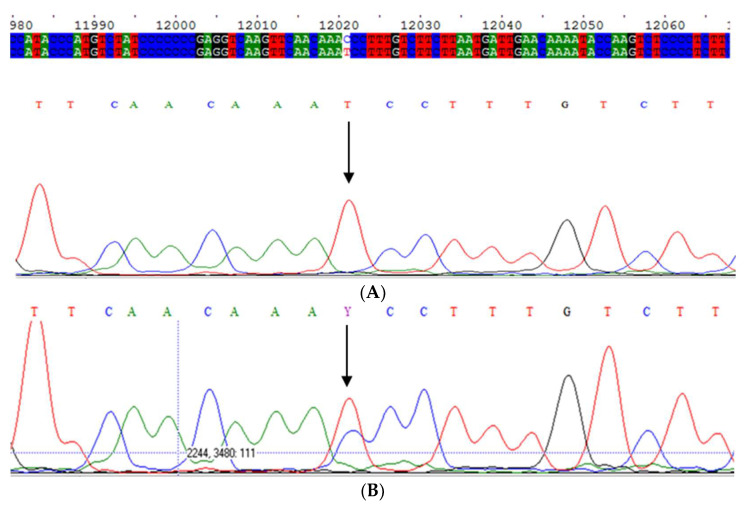

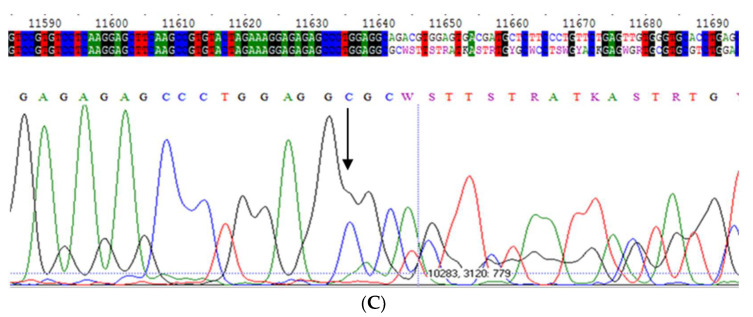
(**A**) Electropherogram of M_wurzuburg_ variant in homozygosis; (**B**) Electropherogram of M_wurzuburg_ variant in heterozygosis; (**C**) Electropherogram of M_whitstable_ variant in heterozygosis.

Subsequently, we selected 18 patients displaying the M_wurzburg,_ IEF pattern from all samples in our archives that had not been sequenced using the current diagnostic algorithm [6], mainly because AAT concentrations were above the decisional cut-off values. We then checked them for the presence of the M_wurzburg_ allele by sequencing exon 5 of the *SERPINA1* gene (Figure 2).

Sixteen of the eighteen subjects (88.9%) were found to carry the M_wurzburg_ variant (Table 1).

Interestingly, one subject had already proven to be S heterozygous and thus had a final PI*SM_wurzburg_ genotype. All other subjects had the PI*MM_wurzburg_ genotype.

Seven of the sixteen reported lung disease patients (43.75%) and one subject (6.25%) also had liver disease.

### 2.2. M_whitstable_

The M_whitstable_ allele consists of: (a) g.11640del26bp, insGG in Sanger annotation [9]; (b) c.1066-262_1066-246delAGTGACGATGCTCTTCC rs553862825 and c.1066-271_1066-265delCAGACGT rs535461370 in Next Generation Sequencing (NGS) annotation (BaseSpace Annotation; Engine 3.6.2.0; Variant Caller: GATK 1.6, Illumina, San Diego, CA, USA).

During routine diagnoses [6] of AATD at Centre for Diagnosis of Inherited Alpha-1 Antitrypsin Deficiency in Pavia, Italy (http://www.alfa1antitripsina.it; accessed on 3 August 2022) from January 2015 to December 2019, we identified the M_whitstable_ allele by intron 4 sequencing in 36 out of 7885 subjects (0.46%), 30 of whom were PI*MM_whitstable_, 5 PI*ZM_whitstable_, and 1 PI*SM_whitstable_ (Table 2).

We retrospectively selected 31 samples analyzed at Centre for Diagnosis of Inherited Alpha-1 Antitrypsin Deficiency in Pavia, Italy, in January 2015, prior the introduction of the intron sequencing into the diagnostic algorithm [6]. The samples had the following features: (a) serum levels equal to or below 120 mg/dL and the absence of pathogenic variants by exon sequencing and the presence of the M2 allele; (b) serum levels higher than 120 mg/dL, C reactive protein (CRP) equal to or higher than 0.8 mg/dL, and the absence of pathogenic variants by sequencing the *SERPINA1* gene coding regions and the presence of the M2 allele. The M_whitstable_ allele was identified by intron 4 sequencing of the AAT gene (Figure 2).

The retrospective analysis of the 31 samples detected the M_whitstable_ variant in 3 subjects (Table 2).

The mean value of AAT serum levels found in patients with the rare M_whitstable_ allele was 87.5 mg/dL, which corresponds to an intermediate AAT deficiency. However, two PI*MM_whitstable_ patients (patients # 2 and #3 in Table 2) displayed lower than expected AAT serum levels and Sanger sequencing did not detect other variants aside from M_whitstable_. Further studies with Next Generation Sequencing are needed for these samples.

Twenty-seven of the thirty-nine subjects carrying the M_whitstable_ allele reported lung diseases (69.2%). Of these, nine were non-smokers (33.3%), nine were former smokers (33.3%), and six were current smokers (22.2%); no information about smoking habits was available for three patients.

## 3. Discussion

M_wurzburg_ and M_whitstable_ are two M-like pathological variants of AAT, which are hard to diagnose using routine laboratory techniques. IEF phenotyping is not usually capable of identifying AAT M-like variants, and the recent rapid diagnostic method A1AT Genotyping Test (Progenika, Biopharma Derio, Biscay, Spain) [13,14] does not include these variants in its panel. Therefore, only sequence analysis enables their diagnosis. The importance of an accurate identification of M_wurzburg_ and M_whitstable_ variants in samples with suspected AATD is evident.

**Figure 3:**
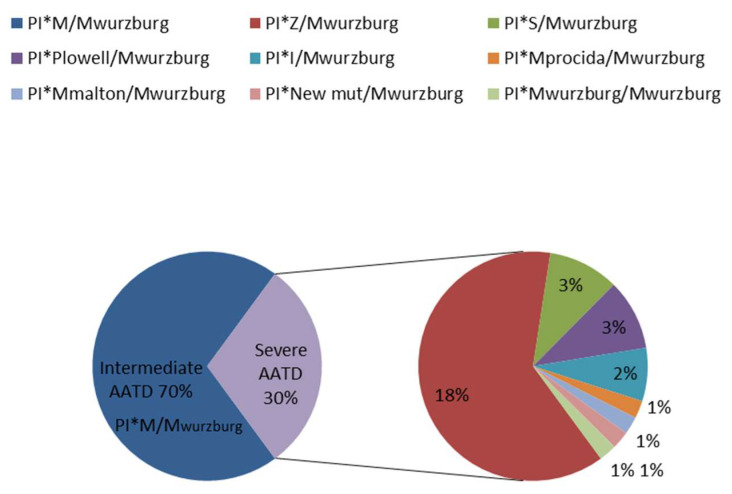
Frequencies of genotypes carrying the M_wurzburg_ allele analysed in the Centre for Diagnosis of Inherited Alpha-1 Antitrypsin Deficiency in Pavia in the last 16 years.

M_wurzburg_ is an AAT pathogenic variant with a tendency to form polymers. Polymerization is the consequence of a transport block between the ER and Golgi complex and it is usually associated with neonatal hepatitis, cirrhosis, and hepatocellular carcinoma. The intracellular polymerization of the M_wurzburg_ protein has been demonstrated in cell models [15,16]. The clinical impact of this variant is not yet clear, since clinical case reports of AATD with the M_wurzburg_ variant are infrequent and controversial [8,17,18]. Nevertheless, in our experience, its presence is rare but not negligible. Accordingly, the allele frequency of M_wurzburg_ in all subjects analyzed in our lab over the last 16 years (for a total of 10,660 samples) is 0.006 (Figure 3).

This value is non-neglectable if we compare it to the frequencies reported in the global (0.000594) and European (0.000642) populations [19].

As shown in Table 1, all samples that present the M_wurzburg_ allele after re-analysis had AAT serum levels above 120 mg/dL, which are normally considered non-pathological concentrations [6]. In our experience, the M_wurzburg_ allele is related to a medium–low AAT concentration, with AAT values lower than the established decisional cut-off (110 mg/dL) [6]. We evaluated a mean AAT of 105.6 mg/dL in the cohort of 94 PI*M/M_wurzburg_ samples diagnosed at Centre for Diagnosis of Inherited Alpha-1 Antitrypsin Deficiency in Pavia. Moreover, the mean AAT value in 25 PI*Z/M_wurzburg_ samples diagnosed at our facility was 53.7 mg/dL, which was in the lower limits of the protective threshold. We should also note that AAT values are occasionally raised not only in individuals with active inflammatory/infectious processes, but also in the event of dehydration, during pregnancy, and in women taking contraceptives [20]. Thus, considering the great importance of an accurate AATD diagnosis related to rare *SERPINA1* variants, the identification of a specific IEF pattern for the M_wurzburg_ variant could prevent the misclassification of samples and make it possible to improve early and accurate AATD detection.

M_whitstable_ is a poorly-studied and pathogenic intronic variant which is not reported in databases. The only available paper in the literature estimated its frequency at 0.0125 in the United Kingdom [9]. Most diagnostic algorithms for the identification of AATD patients include sequence analysis of coding exons by Sanger or Next Generation Sequencing, thus losing the rare variants located within the introns.

According to our series, the variant was evenly distributed in Italy, with a diagnostic rate of 0.002. Most of the PI*M/M_whitstable_ patients that we identified had lung diseases (69.2%) and 82% of subjects reported lung symptoms when questioned on their health status. To underline the role of the M_whitstable_ allele in the lack of lung protection from elastase, we noticed that when smoke was not a co-factor, lung symptoms were reported by 9 out of 11 adult subjects. Interestingly, patients with the M_whitstable_ variant have lower levels of AAT, similar to the MZ population, and thus require a higher level of attention in the diagnostic process.

From a clinical point of view, several patients in our M_whitstable_ cohort were reported to have varying degrees of lung disease, ranging from healthy subjects to those with bronchitis, COPD, or emphysema, regardless of their smoking history in most cases.

The accurate diagnosis of these variants can help the diagnostic process and clinical management, especially in patients with established lung disease without any other reported risk factors. Therefore, it is crucial to refer those patients/samples to expert laboratories, since the IEF or rapid diagnostic tools alone are not usually able to detect those variants. In order to avoid the risk of not identifying patients carrying rare AAT variants, continuous updating of diagnostic algorithms is of chief importance. In this paper, we focused our attention on the identification of two rare M-like AAT variants that are often misdiagnosed. Moreover, for the first time, we have identified the clinical profiles of patients with AATD caused by the M_whitstable_ allele in a fairly large cohort.

## 4. Materials and Methods

### 4.1. Samples

Samples submitted to the Italian Reference laboratory were tested for AATD, following the recently reported diagnostic algorithm [6]. Blood samples from patients were collected in ethylenediamine tetraacetic acid (EDTA) or by using DBS samples (Schleicher & Schuell Grade 903, Keene, New Hampshire, USA) [21].

Informed consent for genetic testing was obtained from all patients according to the institution’s ethical recommendations.

### 4.2. Isoelectrophocusing (IEF)

The phenotype of the M_wurzburg_ variant was obtained by using the semiautomatic Sebia Hydrasys^®^ System and the Hydragel 18 A1AT Isofocusing^®^ kit (Sebia, Diagnostic Department, Evry, France). The procedure consists of sample runs on ready-to-use 0.1% agarose gels that contain ampholytes with a pH gradient ranging from 4.2 to 4.9. After sample migration, the detection of AAT was performed with specific AAT antiserum labelled with peroxidase. The gel was then washed and dried automatically [11,12].

Serum samples and controls were diluted 1:10 in a specific diluent, according to the manufacturer’s instructions. Dried blood spots were cut into 6mm-diameter circles and eluted in 30 µL of water overnight at 4°C. Subsequently, a 1:8 dilution was applied.

### 4.3. Sequencing

We confirmed the presence of the M_wurzburg_ allele by sequencing exon 5 of the SERPINA1 gene. We sequenced the intron 4 region to search for the M_whitstable_ variant.

Polymerase chain reactions (PCR) were performed with the AccuPrimeTM Taq DNA Polymerase System kit (Invitrogen by Thermo Fisher Scientific, Waltham, Massachusetts, USA) and both reaction conditions were as follows: 100 ng of DNA, 2.5 μL of Buffer II, 0.25 μL of MgCl2, 0.5 μM of each primer, and 0.5 μL of Taq DNA Polymerase. Primers used in the amplification and sequence reactions are listed in Table S1. Amplification and sequence reactions were carried out in an I-cycler Thermal Cycler (Bio-Rad Laboratories, Hercules, California, USA). Then, the reaction products were purified with a commercial kit based on magnetic beads (Ampure XP, Inc., Brea CA, USA). Sequence reactions were performed as indicated by the manufacturer, and sequence products were purified by a commercial kit based on magnetic beads (Agencourt CleanSeq, Inc., Brea, CA, USA).

Sequencing was performed by the CEQ 8800 genetic analysis System (Beckman Coulter, Pasadena, California, USA).

The NCBI Reference Sequence which we refer to is the following: NM_001002235.2.

## 5. Conclusions

The identification of a specific IEF pattern amenable to the M_wurzburg_ variant could help detect this pathogenic variant during routine analysis based on phenotyping. Conversely, the sequencing of intron 4 of *SERPINA1* gene enables the identification of M_whitstable_ allele, whose frequency is not neglectable. Since patients carrying the M_wurzburg_ or M_whitstable_ alleles have a higher risk of developing lung and liver diseases or only lung diseases, respectively, an early and precise detection would improve the clinical management of these patients.

## Figures and Tables

**Table 1:** Clinical data of selected and re-examined patients with the M_wurzburg_ IEF pattern.

Sample	Genotype	AAT (mg/dL)	CRP (mg/dL)	Age at Diagnosis	Smoking Habits (Pack/Year)	Disease
1	SM_wurzburg_	86	Not reported	63	Current (31)	Healthy
2	MM_wurzburg_	122.5	0.11	67	Not reported	Emphysema
3	MM_wurzburg_	123	0.11	67	Current (47)	Emphysema
4	MM_wurzburg_	123.2	0.34	25	Not reported	Not reported
5	MM_wurzburg_	124	0.32	64	Former (30)	Asthma
6	MM_wurzburg_	125	0.15	61	Former (16)	Bronchiectasis
7	MM_wurzburg_	125.2	0.28	27	Not reported	Not reported
8	MM_wurzburg_	126	0.05	23	Not reported	Not reported
9	MM_wurzburg_	126	0.14	68	Never	Not reported
10	MM_wurzburg_	131.6	0.06	77	Not reported	Not reported
11	MM_wurzburg_	146.3	0.62	74	Not reported	Healthy
12	MM_wurzburg_	177.2	0.2	55	Never	Asthma, bronchiectasis, dyspnea—hepatitis
13	MM_wurzburg_	180.5	0.11	45	Not reported	Not reported
14	MM	182	0.25	78	Never	Bronchiectasis
15	MM	184	Not reported	48	Not reported	Healthy
16	MM_wurzburg_	197.4	0.05	55	Not reported	Emphysema
17	MM_wurzburg_	206	3.27	78	Not reported	Not reported
18	MM_wurzburg_	315.1	0.09	64	Former (30)	Bronchitis

**Table 2:** Clinical data of patients carrying the M_whitstable_ rare allele.

Sample	Genotype	AAT (mg/dL)	CRP (mg/dL)	Age at Diagnosis	Smoking Habits (Pack/Year)	Disease
1	ZM_whitstable_	48	0.1	64	Never	Not reported
2	M_2_M_whitstable_	55.7	0.1	60	Not reported	Not reported
3	M_2_M_whitstable_	56	0.1	70	Never	Emphysema
4	ZM_whitstable_	56.2	0.11	58	Never	Hepatitis
5	ZM_whitstable_	59.9	0.03	25	Never	Healthy
6	ZM_whitstable_	60.6	0.01	35	Never	Asthma
7	ZM_whitstable_	62	1.4	85	Former (18)	Not reported
8	M_2_M_whitstable_	66	0.1	55	Former (10)	Bronchitis,
9	M_1_M_whitstable_	66.3	0.28	76	Former (6.3)	Asthma, bronchitis, bronchiectasis
10	M_1_M_whitstable_	68.4	0.6	73	Former (18)	Bronchitis
11	M_1_M_whitstable_	70.7	0.2	64	Never	Dyspnea-hepatitis
12	M_2_M_whitstable_	72.4	0.2	46	Never	Emphysema, pneumothorax
13	M_1_M_whitstable_	74	Not reported	Not reported	Not reported	Healthy
14	M_1_M_withstable_	77.1	0.1	38	Not reported	Chronic bronchitis, allergic asthma
15	SM_whitstable_	78	0	15	Never	Not reported
16	M_1_M_whitstable_	78.3	0.06	55	Former (38)	Asthma
17	M_1_M_withstable_	82.1	0.01	47	Not reported	Not reported
18	M_1_M_whitstable_	84.5	0.23	53	Never	Asthma
19	M_2_M_whitstable_	86	0.1	10 months	Never	Bronchitis
20	M_1_M_whitstable_	87.5	0.1	24	Never	Bronchiectasis
21	M_3_M_whitstable_	88	0.1	67	Current (35)	Bronchiectasis, dyspnea, emphysema, bronchitis-hepatitis
22	MM_whitstable_	89.3	0.4	61	Current (45)	Dyspnea
23	M_3_M_whitstable_	89.8	0.13	71	Former	Asthma
24	M_1_M_whitstable_	89.9	0.004	64	Current (31)	Emphysema-hepatitis
25	M_1_M_whitstable_	90	0.13	47	Never	Asthma, emphysema
26	M_1_M_whitstable_	90.7	0.1	66	Never	Bronchiectasis
27	M_1_M_whitstable_	91	Not reported	62	Former (40)	Emphysema
28	M_2_M_whitstable_	91.7	0.01	54	Former (Not reported)	Bronchitis
29	M_1_M_whitstable_	92.9	0	37	Not reported	Not reported
30	M_1_M_whitstable_	93.3	0.1	73	Not reported	Emphysema, pulmonary fibrosis
31	M_3_M_whitstable_	93.7	0.03	3	Never	Healthy
32	M_2_M_whitstable_	94.6	0.12	50	Current (12)	Emphysema
33	M_2_M_whitstable_	96	0.2	55	Current (40)	Emphysema
34	MM_whitstable_	99.6	0.13	27	Current (7.4)	Healthy
35	M_1_M_withstable_	103.9	0.83	72	Not reported	COPD, emphysema
36	M_3_M_whitstable_	104	1	64	Former (Not reported)	Emphysema
37	M_1_M_whitstable_	111.4	0.9	72	Former (0.25)	Asthma, bronchiectasis, dyspnea
38	M_2_M_whitstable_	125	0.05	56	Current (45)	Bronchitis, emphysema, dyspnea
39	MM_whitstable_	128.1	0.12	31	Current (14)	Healthy

## Data Availability

De-identified data are available upon request by contacting the corresponding author (s.ottaviani@smatteo.pv.it).

## Supplementary Materials

The following supporting information can be downloaded at: https://www.mdpi.com/article/10.3390/ijms23179859/s1.
