# Supplementary material for: Improving the Laboratory Diagnosis of M-like Variants Related to Alpha1-Antitrypsin Deficiency
Source: Int J Mol Sci. 2022 Aug 30;23(17):9859. doi: 10.3390/ijms23179859 (PMC9456480; doi:10.3390/ijms23179859)
Supplement: Supplementary file 1 [file ijms-23-09859-s001.zip › ijms-1843220-supplementary.pdf]

Amplified region	Primer	Sequence
exon 5	F	5’-GAGCCTTGCTCGAGGCCTGGGATC-3’
exon 5	R	5’-CAGAGAAAACATGGGAGGGATTTACA-3’
exon 5	a1-5F	5’-CCTGGGATCAGCCTTACAAC-3’
intron 4	F	5’-GATCACCCTGACGACCTTGT-3’
intron 4	R	5’-GGGAGAGAAGCAGAGACACG-3’

Table S1. Primers used both for amplification and sequence reactions.
